# Development and Validation of a Japanese-Language Questionnaire to Screen for Tension-Type Headaches and Migraines

**DOI:** 10.7759/cureus.44633

**Published:** 2023-09-04

**Authors:** Kaho Tanobe, Minori Machida, Ryo Motoya, Atsushi Takeoka, Daisuke Danno, Junichi Miyahara, Takao Takeshima, Hiroaki Kumano, Jun Tayama

**Affiliations:** 1 Psychology, Graduate School of Human Sciences, Waseda University, Saitama, JPN; 2 Clinical Psychology, Graduate School of Human Sciences, Waseda University, Saitama, JPN; 3 Clinical Psychology, School of Psychological Science, Health Sciences University of Hokkaido, Hokkaido, JPN; 4 Health Sciences, Center for Health and Community Medicine, Nagasaki University, Nagasaki, JPN; 5 Headache Center, Department of Neurology, Tominaga Hospital, Osaka, JPN; 6 Behavioral Medicine, Faculty of Human Sciences, Waseda University, Saitama, JPN; 7 Clinical Psychology, Faculty of Human Sciences, Waseda University, Saitama, JPN

**Keywords:** tension-type headache, migraine, validity, questionnaire, sensitivity, specificity, screening

## Abstract

Introduction

Migraine and tension-type headache (TTH) are chronic diseases associated with significant socioeconomic losses and social and psychological impact (current global prevalence: 10% and 38%, respectively). Thus, they require accurate identification and classification. In clinical practice, validated screening tools able to quickly determine migraine and TTH with high sensitivity and specificity help provide an objective and multifaceted understanding of patients' headache symptoms. However, no tool has been developed or validated yet in Japan to ask multifaceted questions about headache-related symptoms in order to identify migraine and TTH and understand these symptoms. This study aimed to develop a questionnaire for screening TTH and migraine.

Methods

The study was conducted from March to June 2022 at a medical institution in Osaka, Japan. The questionnaire - comprising 24 questions that were generated based on the 3rd edition of the International Classification of Headache Disorders - was used to screen for migraine and TTH, aiming for a deeper understanding of related symptoms. The participants were patients aged ≥18 years with at least one of the following diagnoses: migraine, TTH. The participants were asked to respond in writing or online. The inclusion criteria were age ≥18 years; headache patients attending a hospital; and diagnoses of at least one of the following: migraine, TTH. The informativeness and discriminating ability of the screening items were evaluated using the item response theory. Items with a calculated discrimination ≥1.35 (high or very high) were retained for screening purposes. Basic questions required to screen for primary headaches were retained, despite their limited computational discrimination power. Ultimately, nine and eight screening items were finalized for migraine and TTH, respectively. The previous neurologists' clinical diagnosis of each patient was used as the gold standard reference for calculating sensitivity, specificity, and positive and negative predictive values regarding the screening items. Cohen's kappa coefficients with 95% CIs were also calculated to determine the agreement between the neurologists' clinical diagnosis and the questionnaire results.

Results

The study population comprised 69 patients aged 19-89 years who were assisted at a hospital division specializing in headache medicine and diagnosed by neurologists. Of these, 22 patients had migraine, 30 had TTH, and 17 had migraine/TTH. Comparing the neurologists’ clinical diagnosis with our screening questionnaire results, the sensitivity and specificity were 72.7% and 86.7% for migraine and 50.0% and 86.4% for TTH, respectively.

Conclusions

Our brief screening tool was highly specific for diagnosing migraine and TTH in individuals with headache symptoms but lacked sufficient sensitivity, especially for TTH. The high specificity for migraine and TTH suggests that the screening tool we developed in this study can correctly identify those who do not have migraine and TTH. The sensitivity was also relatively high for migraine, suggesting that the tool can correctly identify migraine-positive individuals. However, the sensitivity for TTH was low. This tool could help clinicians in providing detailed course assessment of migraine symptoms and TTH symptoms; however, the issue of low sensitivity for TTH needs to be addressed.

## Introduction

Headache is the general term for pain that occurs in part or all of the head. Like fever and abdominal pain, headache is the name of a symptom, but chronic recurrent headache attacks are treated as headache disorders [1]. Primary headache is a condition in which the headache itself is the primary symptom. Migraine and tension-type headache (TTH) are the most common primary headaches. A headache is treated as a headache disorder when the patient has recurrent headache attacks with similar symptoms. There are various criteria for treatment as a headache disorder, including the frequency of headaches, duration of a single headache, pain intensity, nature of pain, and presence of symptoms such as nausea associated with pain. Both migraine and TTH have subtypes, and specific diagnostic criteria differ for each subtype [1].

Typical characteristics of migraine headaches are a unilateral location, pulsating quality, moderate or severe intensity, aggravation by routine physical activity, and association with nausea and/or photophobia and phonophobia. Typical characteristics of TTH include bilateral pain of pressing or tightening quality and mild to moderate intensity [1].

The considerable prevalence of TTH and migraines has been calculated in numerous studies using various methods including personal or telephonic interviews and self-administered questionnaires. These methods are based on headache diagnostic criteria, including The Headache Classification Committee of the International Headache Society (IHS) [2,3]. The global migraine and TTH prevalence were calculated from 107 epidemiological studies, including 48 from Europe, 20 from Asia, 14 from North America, 13 from Central/South America, eight from Africa, and four from Australia/Oceania [2]. The results found that the global prevalence of migraines and TTHs was 10% and 38%, respectively. Among adults, the reported lifetime migraine prevalence is 11%, and the current TTH prevalence is 42% [2]. Moreover, the lifetime migraine and TTH prevalence is 14% and 46%, respectively [2]. In Japan, both of migraine and TTH prevalence studies and a nationwide survey have been conducted [4, 5], calculating prevalence rates using telephonic interviews and questionnaires based on the International Headache Society (IHS) classification. These studies included 4029 participants (men: 48.7%, women: 51.3%) aged 15 years or older and found that the prevalence of TTH and migraine were 22.4% and 8.4%, respectively [4]. In a population-based survey conducted among all residents aged 20 years or older in the rural western Japanese community of Daisen (N = 5758; 2681 men and 3077 women), questionnaires and telephonic surveys based on IHS diagnostic criteria were used; this survey found that 21.7% residents had TTHs and 6.0% had migraine headaches [5].

According to the Global Burden of Diseases, Injuries, and Risk Factors (GBD) study, migraine and TTH rank the second and ninth highest in age-standardized disability-adjusted life-year rates for all neurological disorders, respectively [6]. A study that examined the association between headache symptoms and disability and self-reported health status among young adults reported that those with migraines and TTH [7] had significantly more physical pain, less vitality, poorer social functioning, and poorer mental health than those without headaches.

Additionally, TTH and migraines cause significant socioeconomic losses. According to Pop et al., the cost for individuals associated with lost labor days due to migraine in the previous four weeks was estimated to be 3.565 US$, whereas loss of productivity was estimated to cost an additional 5.431 US$ [8]. In TTH also, the cost for individuals for lost labor days due to TTH in the previous four weeks was estimated to be 1.523 US$, whereas loss of productivity cost another 2.795 US$ [8].

With such common and burdensome conditions, migraine and TTH should be treated appropriately and not left untreated as much as possible, for which these headaches need to be correctly recognized and classified. Biomarkers are useful in correctly identifying the disease in many conditions. However, there are no established biomarkers that are useful in the diagnosis of migraine and TTH [9, 10]. Since there are no biological markers, primary headache in epidemiologic studies can only be diagnosed through a precise clinical interview conducted according to the current diagnostic criteria of the International Classification of Headache Disorders (ICHD) [11].

Several countries have developed screening tools in their respective languages that can quickly determine migraine and TTH with high sensitivity and specificity [11-24]. Such validated screening tools are helpful in clinical practice because they provide an objective and multifaceted understanding of the symptoms of headache patients.

Furthermore, migraine and TTH symptoms are expected to vary among patients [1]. Even for the same category of headache, patients may have different problems because of the severity of their symptoms and the nature of their pain. Thus, there is a need for a tool that combines the ability to identify a patient's headache type with the ability to ask patients about pain symptoms from multiple perspectives. However, no tool has been developed or validated yet in Japan to ask patients multifaceted questions about their headache symptoms in order to identify migraine and TTH and understand their clinical symptoms. Moreover, few tools have used the ICHD-3 classification diagnostic criteria to design and validate headache types [24] and there are none in the Japanese language. Therefore, there is a need to develop a screening tool in the Japanese language that can be used in clinical practice based on the criteria of the International Classification of Headache.

The aim of this study is to develop a questionnaire for screening TTH and migraine. The procedure was performed with headache specialists and designed to better understand patients’ headache symptoms. The questionnaire was developed based on the ICHD-3 [1], and its validity was assessed using screening items for each headache type.

This article was previously presented as a meeting abstract at the 2022 Japan Society of Stress Management Meeting on November 5-6, 2022, and the 2022 Institute of Applied Brain Sciences Waseda University Symposium on February 27, 2022. Additionally, this article was previously posted to the Research Square preprint server on December 4, 2022.

## Materials and methods

The procedure was performed with the help of headache specialists and was designed to better understand patients’ headache symptoms. The study was registered at the University Hospital Medical Information Network (UMIN) under the registration number UMIN000047197. Several items in the questionnaire were validated to ensure they adequately screened for these types of headaches. This research plan was conducted according to the “Consensus-based Standards for the Selection of Health Measurement Instruments” guidelines [25, 26], and the research tool was created based on the diagnostic criteria (ICHD-3) [1]. ICHD-3 consists of the same items for men and women. There are gender differences in the prevalence of headaches [2], and there may also be other gender differences that influence screening decisions. However, as a first step in creating a tool to correctly determine headache nature, as the diagnostic criteria for headaches are the same for men and women, this study aimed to develop a common screening tool for both genders. However, notably, gender-specific analyses could not be performed in this study owing to the small number of male participants.

Questionnaire structure

The questionnaire included 24 questions that satisfied the ICHD-3 diagnostic criteria for migraine (codes 1.1, 1.2, and 1.3) and TTH (codes 2.1, 2.2, and 2.3). First, two authors (first and last authors) drafted the questionnaire based on ICHD-3 [1]. The first draft consisted of a total of 25 questions according to the diagnostic criteria. These included questions to check the presence of headache, frequency of headache, intensity of pain, duration of illness, use of medication, type of medication, and headache symptoms (nausea, etc.).

Then, they discussed with a specialist in general medicine (fourth author) and a scientist and psychiatry specialist (eighth author) whether this questionnaire correctly reflected the ICHD-3 diagnostic criteria. Then, they added and modified questions with the help of three neurologists specializing in headaches (fifth, sixth, and seventh authors). The specific changes made are as follows:

Questions that asked patients to freely describe the medication name and whether the medication was over-the-counter or by prescription were deleted because they were considered to be irrelevant to the diagnosis of headaches. For the same reason, questions on the degree of nausea at the time of the headache were also deleted. In the question about the type of pain, the terminology "pulsating quality or not" for migraine was replaced with the more easily understood phrase "Throbbing pain that feels like it is in time with my pulse." Additionally, for TTH, the question "Do you have a tightening pain?" was changed to "Heavy pain that feels as if it is squeezing." Other changes were made to make the questionnaire less burdensome for patients, such as formulating the items checking for the presence of aura and on the duration of headache as “yes/no” questions and selecting all the applicable choices. We also added a question to confirm that the patient had recurrent headaches of the same type, which is a feature of migraine and TTH.

**Figure 1:**
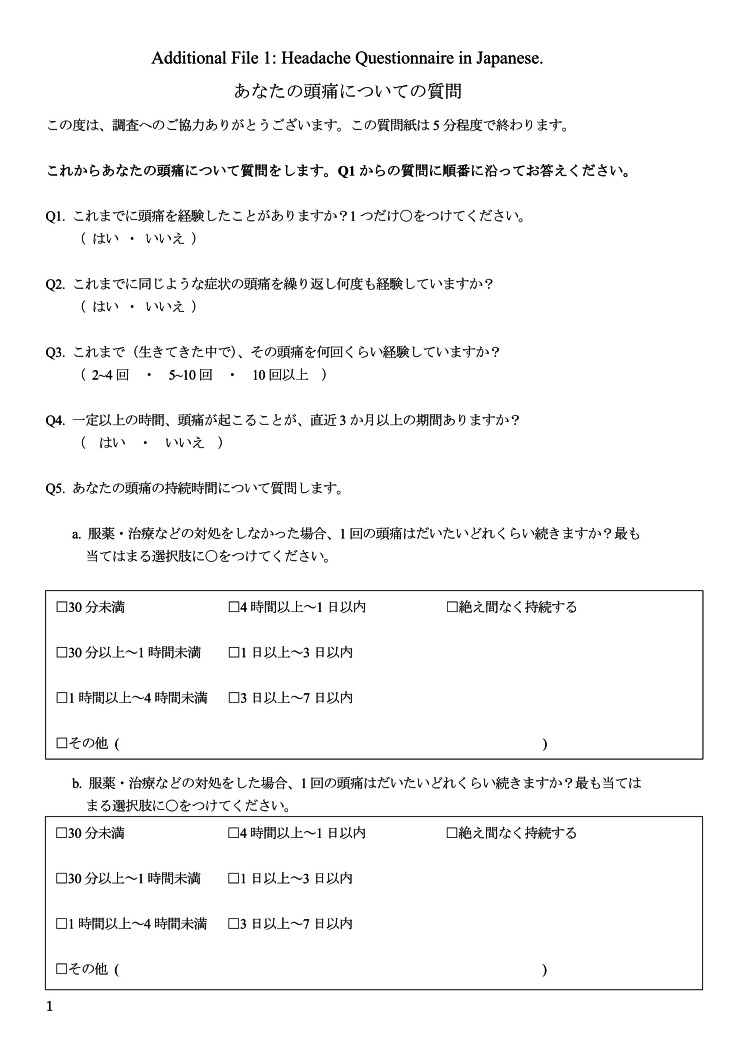
Headache Questionnaire in Japanese: page 1.

**Figure 2:**
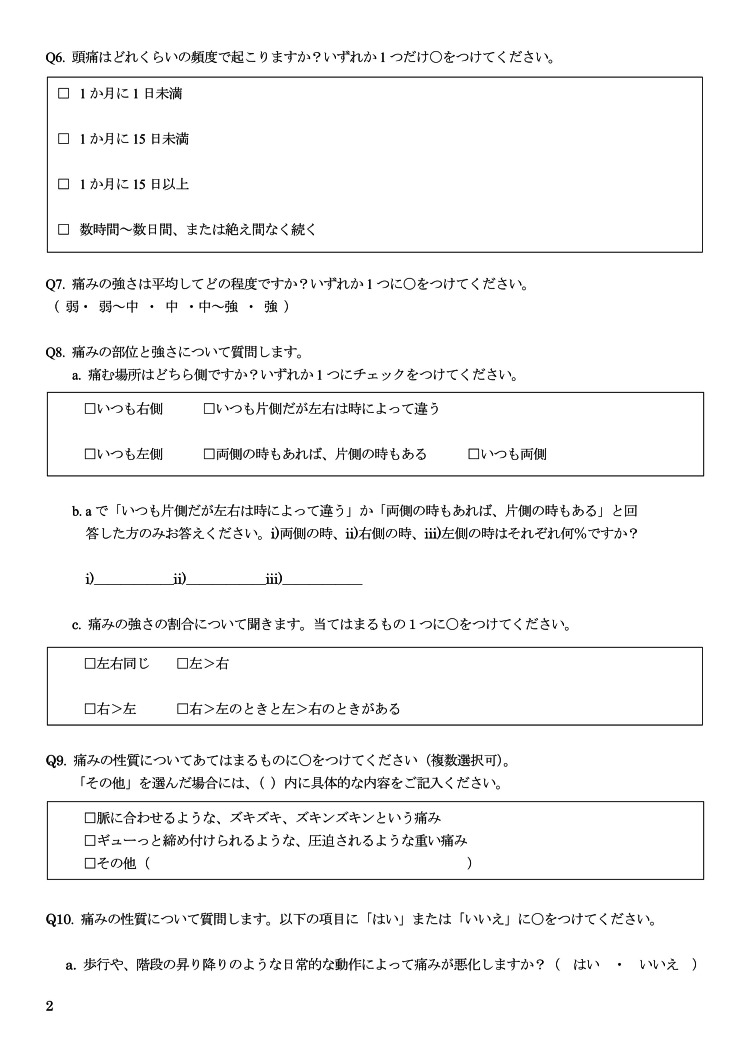
Headache Questionnaire in Japanese: page 2.

**Figure 3:**
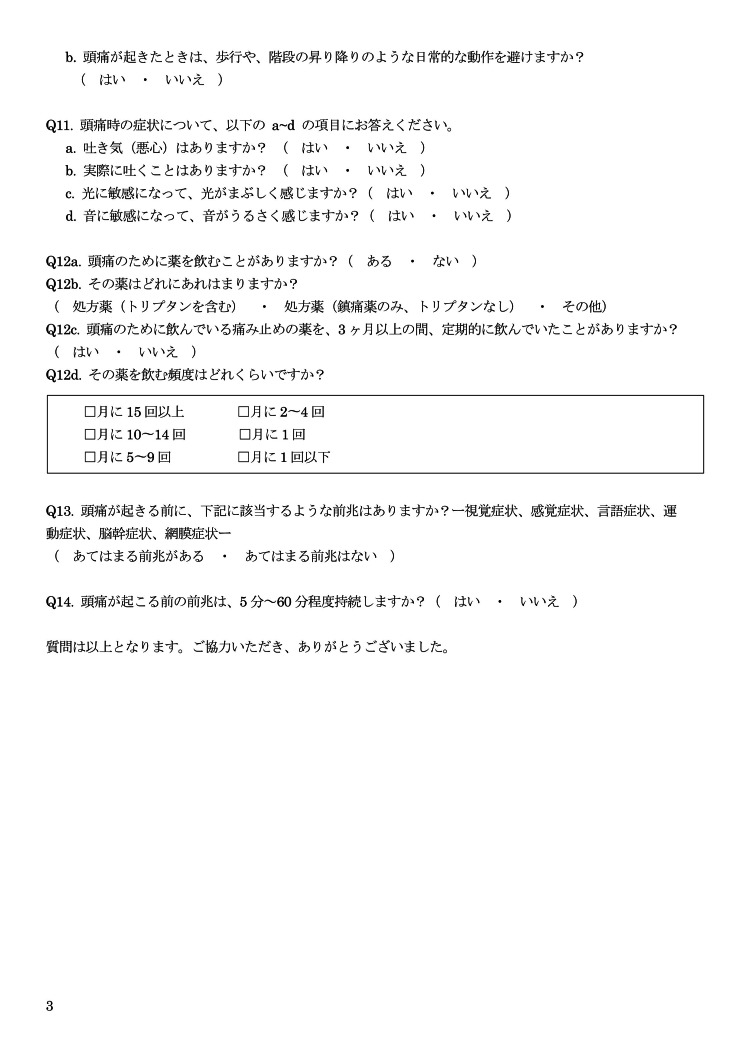
Headache Questionnaire in Japanese: page 3.

**Figure 4:**
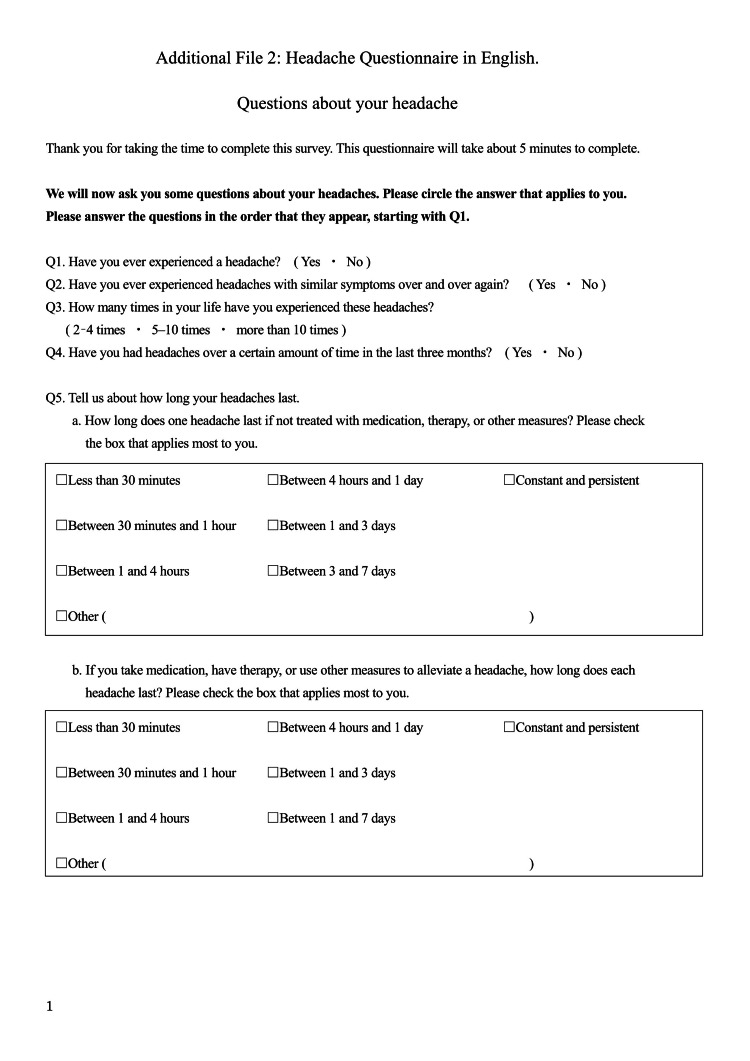
Headache Questionnaire in English: page 1.

**Figure 5:**
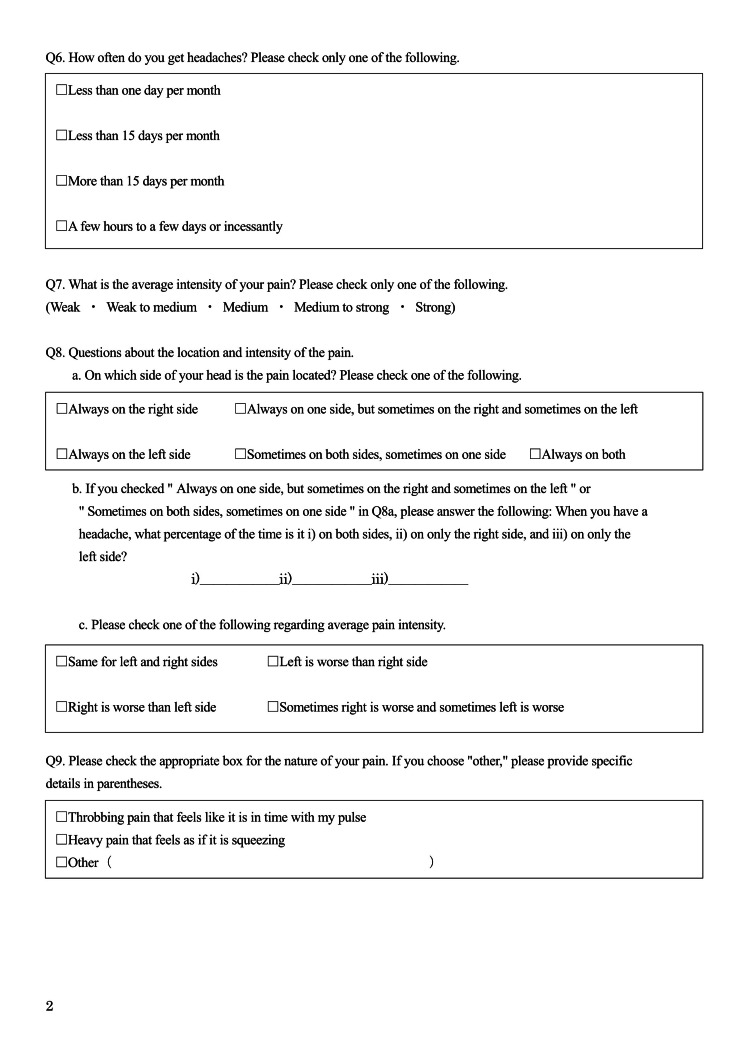
Headache Questionnaire in English: page 2.

**Figure 6:**
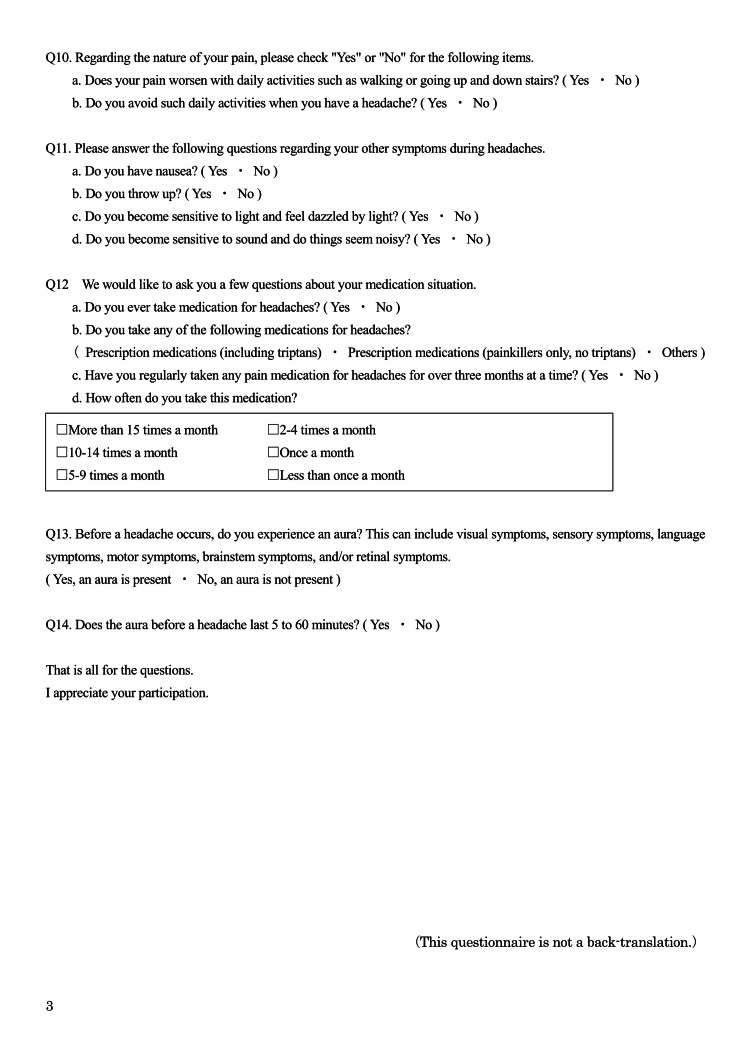
Headache Questionnaire in English: page 3.

Thus, the final 24 items were decided (Figures 1-3 in the Appendix). Although the questionnaire developed for this study was in Japanese, an English version is also provided in this paper (Figures 4-6 in the Appendix). The questionnaire comprises items to confirm the presence or absence of chronic headache (Q1, Q2, Q3, and Q4), others to screen for migraine and TTH (Q7, Q9, Q10ab, Q11abcd, Q12ab, Q13, and Q14), and questions for a deeper understanding of the patient's symptoms (Q5ab, Q6, Q8abc, and Q12cd).

The responses to questions varied according to the following types: (1) "Yes" or "No" (e.g., as in a reply to "Does your pain worsen with daily activities, such as walking or going up and down stairs?"); and (2) single-choice (e.g., "How often do you get headaches? Please check only one of the following."); and (3) multiple-choice (e.g., Please check the appropriate box for the nature of your pain.); and (4) open-ended response (e.g., If you checked "Always on one side, but sometimes on the right and sometimes on the left" or "Sometimes on both sides, sometimes on one side" in Q8a, please answer the following: When you have a headache, what percentage of the time is it i) on both sides, ii) on only the right side, and iii) on only the left side?). Details on the questions are presented in the Appendix. In addition to this questionnaire, basic information on age and gender was also collected.

In total, 10 items were used to screen for migraine (Q7, Q9, Q10ab, Q11abcd, and Q12ab) and 11 for TTH (Q7, Q9, Q10ab, Q11abcd, Q12a, Q13, and Q14). These screening items were designed to meet the diagnostic criteria of the ICHD-3 based on the core symptoms of migraine and TTH, respectively.

Study participants

All study participants had a previous clinical diagnosis. The inclusion criteria were (1) age ≥18 years and (2) headache patients attending a hospital, and (3) patients with at least one of the following diagnoses: migraine, TTH, migraine/TTH. The exclusion criteria were (1) age <18 years.

Survey period and institution

The survey was conducted from March to June 2022 at the Department of Headache Neurology, Kotobuki-kai Tominaga Hospital, Osaka, Japan.

Study flow

A set of documents explaining participation in the study and a questionnaire prepared by the first author were distributed to the patients by the fifth, sixth, and seventh authors, all of whom are neurologists, at the patients’ office visits. Patients who indicated their willingness to participate in the study in these documents were asked in writing to answer the questions. Patients were asked to respond to the survey using their preferred method: filling out the paper questionnaire or completing the online version via a QR code provided with the instructions.

Selection of screening items using IRT

The data were analyzed with JMP software program (JMP® Pro, Version 16.2.0; SAS Institute Inc., Cary, NC, USA). A two-parameter (2-PL) logistic Item Response Theory (IRT) model was developed to evaluate each screening item for its informativeness (discrimination).

Among the items required to screen for migraine and TTH (Q7, Q9, Q10ab, Q11abcd, Q13, and Q14), those with a calculated discrimination ≥1.35 (high or very high) were retained as screening (migraine: Q10ab and Q11abcd; TTH: Q10a, Q11abcd, and Q13) [27]. Other items (migraine: Q1 and Q2, Q12a; TTH: Q1 and Q3) were also retained as screening, being basic questions needed to check the diagnosis of primary headache, though they had low computational discrimination power. As a result, the final screening items were nine in number for migraine and eight for TTH.

Calculation of sensitivity, specificity, and positive/negative predictive values

After conducting a sample size test, we determined that data from at least 20 individuals would be required to ensure an accuracy of 80% sensitivity and specificity [28, 29]. The sensitivity, specificity, and positive and negative predictive values were calculated for migraine, TTH. The 95% confidence intervals (CI) for each statistic were calculated using the Agresti-Coull method. The neurologists’ clinical diagnosis (clinical diagnosis previously issued to the patient) was used as the gold standard reference for calculating each statistic in comparison with the results of the screening questionnaire. Cohen's kappa coefficients with 95% CIs were calculated to determine the agreement between the neurologists’ clinical diagnosis and the questionnaire's results. Statistical analyses were performed using EZR [30], a statistical software application that extends the capabilities of R and R Commander.

Approval of the ethics committee

This study was approved by the Ethics Committee on Research Involving Human Subjects of Waseda University in Japan (approval no., 2021-078) and the Ethics Committee of Kotobuki-kai Tominaga Hospital (approval no., 120116).

## Results

The diagnostic criteria for migraine and TTH according to the 3rd edition of the International Classification of Headache Disorders (ICHD-3) are described in Table 1 and Table 2, respectively [1].

**Table 1:** ICHD-3 criteria for migraine. ICHD: International Classification of Headache Disorders

Migraine (1.1, 1.2, 1.3)
ICHD-3 criteria
1.1. Migraine without aura
A. At least five attacks fulfilling criteria B–D
B. Headache attacks lasting 4–72 hours (when untreated or unsuccessfully treated)
C. Headache has at least two of the following four characteristics:
1. Unilateral location
2. Pulsating quality
3. Moderate or severe pain intensity
4. Aggravation by or causing avoidance of routine physical activity (e.g., walking or climbing stairs)
D. During headache at least one of the following:
1. Nausea and/or vomiting
2. Photophobia and phonophobia
E. Not better accounted for by another ICHD-3 diagnosis.
1.2. Migraine with aura
A. At least two attacks fulfilling criteria B and C
B. One or more of the following fully reversible aura symptoms:
1. Visual
2. Sensory
3. Speech and/or language
4. Motor
5. Brainstem
6. Retinal
C. At least three of the following six characteristics:
1. At least one aura symptom spreads gradually over ≥5 minutes
2. Two or more aura symptoms occur in succession
3. Each individual aura symptom lasts 5–60 minutes
4. At least one aura symptom is unilateral
5. At least one aura symptom is positive
6. The aura is accompanied, or followed within 60 minutes, by headache
D. Not better accounted for by another ICHD-3 diagnosis.
1.3. Chronic migraine
A. Headache (migraine-like or tension-type-like) on ≥15 days/month for >3 months, and fulfilling criteria B and C
B. Occurring in a patient who has had at least five attacks fulfilling criteria B–D for 1.1 Migraine without aura and/or criteria B and C for 1.2 Migraine with aura
C. On ≥8 days/month for >3 months, fulfilling any of the following:
1. Criteria C and D for 1.1 Migraine without aura
2. Criteria B and C for 1.2 Migraine with aura
3. Believed by the patient to be migraine at onset and relieved by a triptan or ergot derivative
D. Not better accounted for by another ICHD-3 diagnosis.

**Table 2:** ICHD-3 criteria for TTH. ICHD: International Classification of Headache Disorders; TTH: Tension-type headache.

2. Tension-type headache (TTH) (2.1, 2.2, 2.3)
ICHD-3 criteria
2.1. Infrequent episodic tension-type headache
A. At least 10 episodes of headache occurring on <1 day/month on average (<12 days/year) and fulfilling criteria B–D
B. Lasting from 30 minutes to seven days
C. At least two of the following four characteristics:
1. Bilateral location
2. Pressing or tightening (non-pulsating) quality
3. Mild or moderate intensity
4. Not aggravated by routine physical activity such as walking or climbing stairs
D. Both of the following:
1. No nausea or vomiting
2. No more than one of photophobia or phonophobia
E. Not better accounted for by another ICHD-3 diagnosis.
2.2. Frequent episodic tension-type headache
A. At least 10 episodes of headache occurring on 1–14 days/month on average for >3 months (≥12 and <180 days/year) and fulfilling criteria B–D
B. Lasting from 30 minutes to seven days
C. At least two of the following four characteristics:
1. Bilateral location
2. Pressing or tightening (non-pulsating) quality
3. Mild or moderate intensity
4. Not aggravated by routine physical activity such as walking or climbing stairs
D. Both of the following:
1. No nausea or vomiting
2. No more than one of photophobia or phonophobia
E. Not better accounted for by another ICHD-3 diagnosis.
2.3. Chronic tension-type headache
A. Headache occurring on ≥15 days/month on average for >3 months (≥180 days/year), fulfilling criteria B–D
B. Lasting hours to days, or unremitting
C. At least two of the following four characteristics:
1. Bilateral location
2. Pressing or tightening (non-pulsating) quality
3. Mild or moderate intensity
4. Not aggravated by routine physical activity such as walking or climbing stairs
D. Both of the following:
1. No more than one of photophobia, phonophobia or mild nausea
2. Neither moderate or severe nausea nor vomiting
E. Not better accounted for by another ICHD-3 diagnosis.

A total of 79 patients completed the survey. Two patients were <18 years old, and two answered “no” to the question, “Have you ever had a headache?” Answering “no” to this question is a patient response error. Moreover, six patients provided incomplete answers. Therefore, 10 patients were excluded.

Finally, the responses of 69 patients (age, 19-89 years; mean age ± standard deviation, 55.0 ± 18.7 years), who were visiting Kotobukai Tominaga Hospital, were used in the statistical analysis. These 69 patients consisted of 16 (23%) men, 51 (74%) women, and 2 (3%) others. According to the neurologists’ diagnoses, the headache types were migraine (n=22), TTH (n=30), and mixed migraine/TTH (n=17). The 22 patients with migraine consisted of 1 (5%) man and 21 (95%) women. The 30 patients with TTH consisted of 11 (37%) men, 18 (60%) women, and 1 (3%) other. The 17 patients with mixed migraine/TTH consisted of 4 (24%) men, 12 (71%) women, and 1 (6%) other. The demographic data of the surveyed population are shown in Table 3.

**Table 3:** Demographic and clinical characteristics of the study population. MIG: Migraine, TTH: Tension-type headache

	Total	MIG	TTH	MIG + TTH
Age: Mean	55	42	67	51
Range	19-89	19-75	23-89	27-72
Gender: Total	69	22	30	17
Men	16	1	11	4
Women	51	21	18	12
Other	2	0	1	1

Sensitivity, specificity, and positive and negative predictive values

The sensitivity and specificity comparing the neurologists’ clinical diagnosis to our screening questionnaire were as follows: for migraine, 72.7% and 86.7%, and for TTH, 50.0% and 86.4%. The kappa coefficients revealed moderate agreement in the case of migraine (κ = 0.60), whereas for TTH the agreement between the neurologists’ clinical diagnosis and the questionnaire’s results was low (κ = 0.34). Table 4 summarizes these results.

**Table 4:** Sensitivity, specificity, and positive (PPV) and negative (NPV) predictive values for migraine, tension-type headache. MIG: Migraine, TTH: Tension-type headache

	N	Sensitivity %	Specificity %	PPV %	NPV %	Kappa
		(95% CI)	(95% CI)	(95% CI)	(95% CI)	(95% CI)
MIG	22	72.7	86.7	80.0	81.3	0.60
		(51.6; 87.1)	(69.7; 95.3)	(57.8; 92.5)	(64.3; 91.5)	(0.38; 0.82)
TTH	30	50.0	86.4	83.3	55.9	0.34
		(33.2; 66.9)	(65.8; 96.1)	(60.0; 95.0)	(39.4; 71.1)	(0.09; 0.59)

## Discussion

This study aimed to develop a headache-screening questionnaire to obtain a deeper understanding of a patient's headache symptoms. It was designed to meet the ICHD-3 diagnostic criteria for migraine and TTH. The headache questionnaire consisted of a total of 24 items and the response types could be one of the following: "Yes" or "No," single-choice, multiple-choice, and open-ended responses. Of the 24 items, nine and eight were screening items for migraine and TTH, respectively. The survey was administered to outpatients. Patients were asked to provide information on gender and age in addition to responding to the headache questionnaire. Sensitivity, specificity, PPV, NPV and kappa coefficients were calculated for 22 patients with migraine and 30 with TTH.

Several brief screening tools have been developed in various countries for migraine and TTH, each comprising approximately 10 questions [13,23]. Therefore, following the lead of previous studies, this survey was also designed to facilitate responses for both migraine and TTH. Several items in the questionnaire were validated to ensure they were adequately screened for TTH and migraine.

The specificity of the screening tool for migraine and TTH was reasonable. We developed screening items as rigorous diagnostic criteria based on discussions with neurologists specializing in headache and diagnostic classification. Many items in our screening tool were designed to exclude patients with even a single “no” response. These strict exclusion criteria may have increased the specificity of the screening items.

The sensitivity of screening for TTH was low. The characteristics of pain and associated symptoms were less likely to be captured than those of migraine. The symptoms of TTH are diverse and vary among individuals [1]. We believe that a contributing factor to the difficulty in screening may be the variability and ambiguity of the TTH symptoms. In fact, the screening accuracy for TTH is likely to be more difficult than for migraine. In many previous studies, sensitivity for TTH was lower than for migraine [11,12,15,17-21]. Several previous studies [11,22,24] that reported high sensitivity in TTH screening focused on TTH subtypes and pain frequency. However, this study excluded some of the questions used to determine pain severity, such as pain intensity and frequency. This was due to the low discriminant value in the selection of items using IRT.

The agreement (kappa coefficient) between the neurologist's headache clinical diagnosis and questionnaire rating was lower than that in previous studies, except for migraine. The kappa coefficients reported in previous studies ranged from 0.42 to 0.88 for migraine and 0.39 to 1.00 for TTH [12,16-20,24].

Using this screening tool, we also correctly ensured the exclusion of secondary headaches to determine migraine and TTH. Secondary headaches are triggered by an illness or physical injury. Dangerous secondary headaches include, for example, headaches attributed to ischemic stroke or attributed to non-traumatic intracranial hemorrhage [1].

The International Classification of Headache, 3rd edition states the following:

“In a number of other conditions that can induce both headache and stroke, such as dissections, cerebral venous thrombosis, giant cell arteritis and central nervous system angiitis, headache is often an initial warning symptom. It is therefore crucial to recognize the association of headache with these disorders in order to diagnose correctly the underlying vascular disease and start appropriate treatment as early as possible, thus preventing potentially devastating neurological consequences. A clue that points to an underlying vascular condition is the onset, usually sudden, of a new headache, so far unknown to the patient. Whenever this occurs, vascular conditions should urgently be looked for [1].”

Therefore, screening for primary headaches requires, first and foremost, attention to the absence of this life-threatening secondary headache. In this screening tool, patients were asked to answer "yes" or "no" to the question, "Have you ever experienced headaches with similar symptoms over and again?" to exclude secondary headaches. Thus, we designed this question expecting patients experiencing recurrent migraine or TTH to answer "yes" to the above question. We then designed the question such that an affirmative answer would indicate either migraine or TTH and a negative response would indicate that the headache was possibly secondary. However, the survey revealed that many individuals with migraine and TTH answered "no" to the above question; this resulted in them being categorized by the screening tool as having secondary headaches. These results significantly differed from the assumption that all patients with migraines and TTH should have answered "yes" to the question, "Have you ever experienced headaches with similar symptoms over and again?"

We attribute the high number of incorrect responses to this question to the fact that the patients had difficulty determining whether each headache they had was the same or different. Migraine and TTH have a wide range of pain symptoms. The intensity of the pain and site may vary from attack to attack [1]. These factors may have caused the patient to be at a loss to determine whether his headache was the same each time. In addition, the screening tool did not provide any description of the secondary headaches, and no additional description of the pain characteristics of secondary headaches was provided. Therefore, it is possible that the question's intent was unclear, and the patient may have had difficulty answering the question.

The study has the following strengths. First, the questionnaire items for migraine and TTH were developed based on the ICHD-3 diagnostic criteria. The ICHD-3 [1] criteria are standard and highly reliable criteria used worldwide. Second, the number of questions was relatively small; many were multiple-choice with “yes” or “no” responses. Questions with limited options are less burdensome for the participants; thus, many symptomatic persons can answer the questions.

There are three limitations to this study. First, the questionnaire was not designed to classify the TTH subtypes. To classify subtypes, items corresponding to the diagnostic criteria would have to be included in our questionnaire. Second, the participants in this study had already been clinically diagnosed by neurologists with headaches prior to participating in this study. Thus, it is likely that they had been educated about headache symptoms and diagnosis. This finding might have allowed for more accurate answers, fewer errors, and a clearer understanding of headache symptoms than potential patients who did not attend the hospital. The accuracy in the present study might have been lower if the questions had been answered by patients who had not attended a hospital or by headache sufferers with symptoms but unaware of their illness. Future research should survey patients who have not attended a clinic and compare the results with physician judgments to reconfirm the accuracy of the present survey. Third, as the participants in this study were limited in number of patients, it was not possible to determine whether this tool may be used to accurately calculate prevalence rates for men and women. However, it is known that there are gender differences in headache prevalence, with women having a higher prevalence than men [2]. Therefore, future research should also focus on prevalence calculations for men and women using this tool.

## Conclusions

The screening tool developed in this study is brief and highly specific for diagnosing migraine and TTH in people with headache symptoms, but has insufficient sensitivity, particularly for TTH. The high specificity for migraine and TTH suggests that the screening tool developed in this study can correctly identify those who do not have migraine and TTH. The sensitivity was also relatively high for migraine, suggesting that the tool can correctly identify individuals with migraine. However, the sensitivity for TTH was low. This tool could help clinicians in providing detailed course assessment of migraine and TTH symptoms. It is expected that this tool will be used in clinical practice in the future to assist clinicians in diagnosing migraine and TTH. However, the issue of low sensitivity for TTH needs to be addressed in future research. Further improvements regarding the inadequate exclusion of secondary headaches triggered by an illness or physical injury and the inability to classify headache subtypes are also required. These issues need to be re-examined to further improve the accuracy of the tool.
